# Predictors of return of spontaneous circulation and survival in in-hospital cardiac arrest: a retrospective study in a single institution

**DOI:** 10.1186/cc14498

**Published:** 2015-03-16

**Authors:** JL Chua, ZM Lin, JH Tan, S Surentheran, M Haris Bin Iskander, B Leong

**Affiliations:** 1 National University of Singapore, Singapore; 2 National University Hospital, Singapore

## Introduction

Despite several large studies concerning out-of-hospital cardiac arrests in recent years, it is not clear whether their in-hospital counterparts have benefited from advances in resuscitation as well as post-resuscitation care.

## Methods

We identified all cases of in-hospital cardiac arrest (IHCA) occurring in the National University Hospital in Singapore from 1 June 2008 to 31 May 2009. Patients for which IHCA occurred but where no resuscitation was attempted were excluded. Key outcomes were classified as primary (survival to discharge) and secondary (return of spontaneous circulation). Additionally, various arrest characteristics were analysed to identify predictive factors for survival to discharge with level of significant set at *P *< 0.05.

## Results

Among 353 unique cases of IHCA analysed, 63 patients (17.8%) had a shockable rhythm (ventricular fibrillation and pulseless ventricular tachycardia) of which 17 (27.0%) survived to discharge. While 290 (82.2%) patients presented with nonshockable rhythm (asystole or pulseless electrical activity), only 32 patients (11%) survived to discharge. For patients who survived to discharge, univariate analysis showed that event location (*P *= 0.016), nationality (*P *= 0.035), paying class (*P *= 0.038), use of ECG monitoring (*P *= 0.048), initial cardiac rhythm (*P *= 0.000) and presence of a house officer (*P *= 0.005) were statistically significant. Multivariate analysis showed that patients with shockable rhythms were 2.52 times more likely to survive but other factors were not significant. For patients who attained ROSC, univariate analysis showed that time of day (*P *= 0.006), event location (*P *= 0.000), and number of adrenaline doses administered (*P *= 0.000) were statistically significant. Multivariate analysis showed that an arrest occurring in the ICU setting was 2.9 times more likely to attain ROSC (95% CI: 1.02 to 5.59, *P *= 0.044).

## Conclusion

The results of this study have described some key predictive factors regarding positive outcomes in IHCA in Singapore. These are vital in understanding important features regarding IHCAs and will aid in developing policies to help improve care and survival in this group of patients.

